# Supplementary material for: Mindfulness Training Associated With Resting-State Electroencephalograms Dynamics in Novice Practitioners via Mindful Breathing and Body-Scan
Source: Front Psychol. 2021 Oct 28;12:748584. doi: 10.3389/fpsyg.2021.748584 (PMC8581621; doi:10.3389/fpsyg.2021.748584)
Supplement: Supplementary file 1 [file Data_Sheet_1.PDF]

# *Supplementary Material*

## 1 Alpha asymmetry analysis

The results of alpha asymmetry posttest pretest *t* test analysis are shown in supplementary figure 1. The analysis method was accord with the suggestion of Isbel et al. (2019). In MBSR group, although there were trends that alpha asymmetry scores increased in ***resting 1***, the ***breathing*** and the ***body-scan*** after MBSR intervention, the differences between posttest and pretest were insignificant (*ps* > .05), neither on Fp1/2 nor F3/4. In the waitlist control group, there were no significant difference found between posttest and pretest in ***resting 1***, ***breathing*** and ***body-scan*** (*ps* > .05), neither on Fp1/2 nor F3/4.

## 2 Supplementary Figures and Tables

**SUPPLEMENTARY FIGURE 1. Alpha asymmetry t-test analysis on resting 1, breathing and body-scan.** MBSR group $n = 17$, waitlist control group $n = 14$. No significant alpha asymmetry difference was found between posttest and pretest.

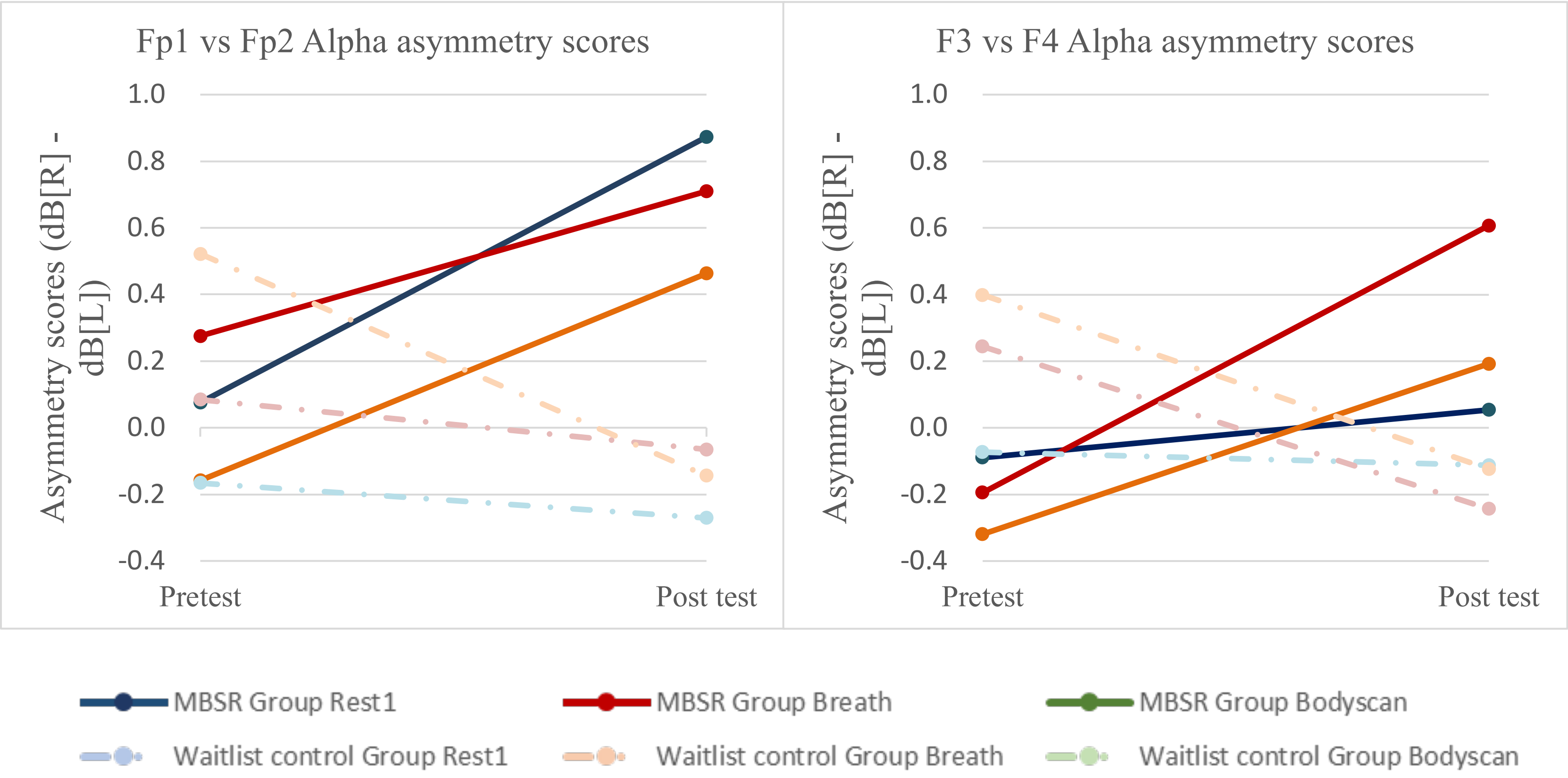

**SUPPLEMENTARY FIGURE 2. The fixation cross image in the scanning sessions.**

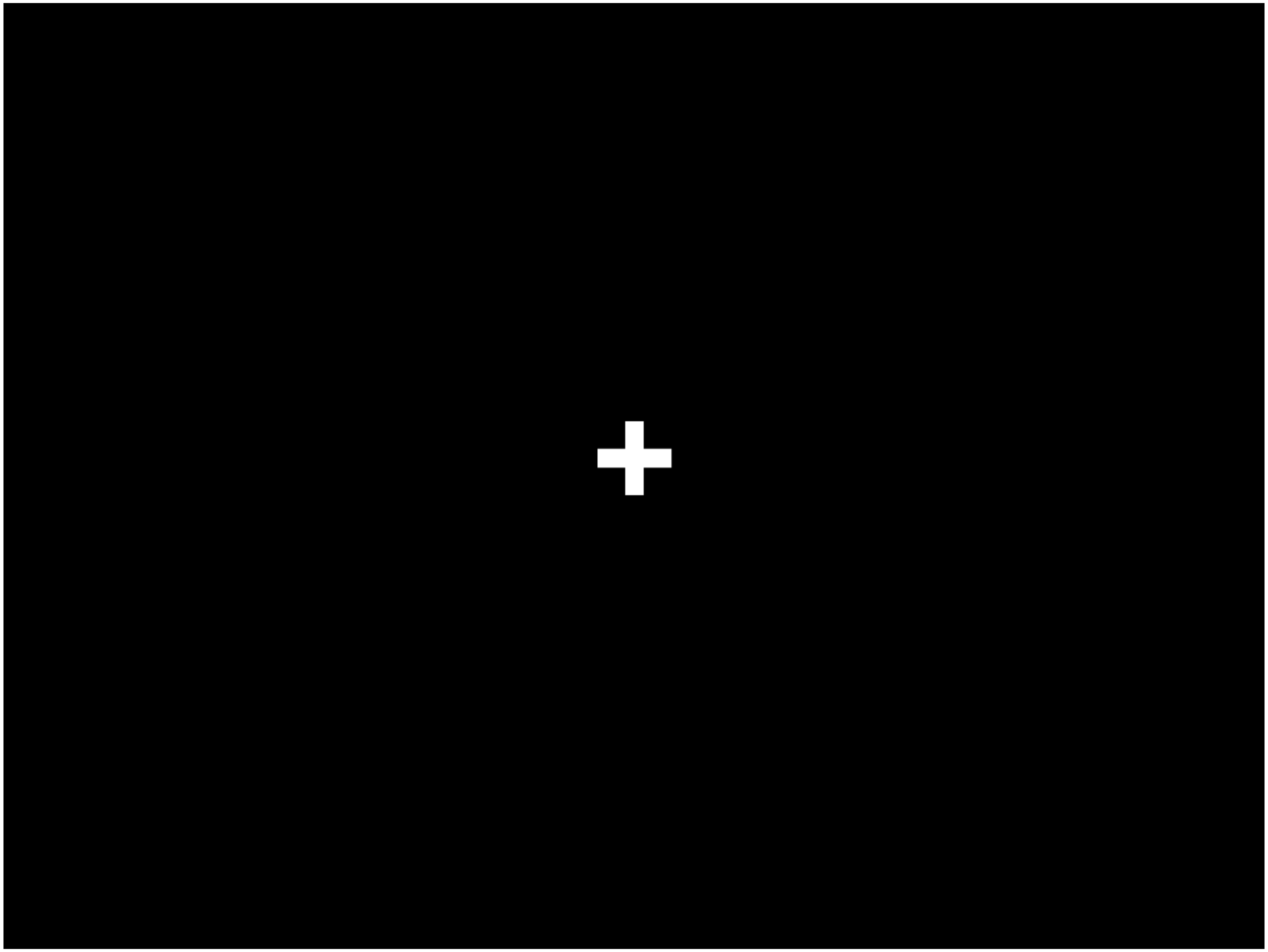

**SUPPLEMENTARY TABLE 1. Correlation between posttest between-task EEG band power difference and behavioral indexes in the MBSR group.** *$p < .05$

| Fz | Breathing - Resting1 | | | | Body-scan - Resting1 | | | |
|---|---|---|---|---|---|---|---|---|
| | FFMQ | | DERS | | FFMQ | | DERS | |
| | r | *p* | r | *p* | r | *p* | r | *p* |
| Delta | -0.08 | 0.747 | 0.03 | 0.895 | -0.35 | 0.166 | 0.33 | 0.199 |
| Theta | 0.04 | 0.878 | -0.25 | 0.324 | -0.23 | 0.371 | 0.12 | 0.636 |
| Alpha | 0.10 | 0.708 | -0.22 | 0.387 | -0.11 | 0.677 | 0.04 | 0.880 |
| Low Beta | -0.15 | 0.559 | -0.06 | 0.832 | -0.38 | 0.135 | 0.26 | 0.319 |
| High Beta | 0.11 | 0.684 | -0.18 | 0.481 | 0.11 | 0.680 | -0.16 | 0.535 |
| Low-gamma | **-0.57*** | 0.018 | **0.69*** | 0.002 | -0.01 | 0.956 | 0.00 | 0.997 |
| **Pz** | **Breathing - Resting1** | | | | **Body-scan - Resting1** | | | |
| | FFMQ | | DERS | | FFMQ | | DERS | |
| | r | *p* | r | *p* | r | *p* | r | *p* |
| Delta | -0.30 | 0.245 | 0.16 | 0.549 | 0.12 | 0.658 | 0.12 | 0.650 |
| Theta | -0.19 | 0.457 | -0.05 | 0.844 | 0.11 | 0.679 | 0.02 | 0.952 |
| Alpha | -0.02 | 0.936 | -0.09 | 0.742 | 0.41 | 0.099 | -0.22 | 0.400 |
| Low Beta | -0.20 | 0.443 | 0.09 | 0.726 | 0.18 | 0.485 | 0.06 | 0.827 |
| High Beta | -0.41 | 0.104 | 0.31 | 0.226 | 0.19 | 0.470 | 0.00 | 0.999 |
| Low-gamma | -0.40 | 0.111 | 0.46 | 0.061 | -0.10 | 0.713 | 0.29 | 0.265 |

**SUPPLEMENTARY TABLE 2. Correlation coefficients among the power changes of EEG wave bands and FFMQ subscales in MBSR group.** *$p < .05$, **$p < .01$

| | | Fz | | | | | | Pz | | | | | |
|---|---|---|---|---|---|---|---|---|---|---|---|---|---|
| | | Δdelta | Δtheta | Δalpha | Δlow-beta | Δhigh-beta | Δlow-gamma | Δdelta | Δtheta | Δalpha | Δlow-beta | Δhigh-beta | Δlow-gamma |
| Resting 1 | ΔFFMQ_observing | 0.38 | 0.50* | 0.23 | 0.47 | 0.44 | 0.36 | 0.20 | 0.06 | -0.04 | 0.34 | 0.39 | 0.30 |
| | ΔFFMQ_describling | 0.41 | 0.49* | 0.23 | 0.28 | -0.04 | 0.02 | 0.39 | 0.27 | 0.32 | 0.35 | 0.08 | -0.02 |
| | ΔFFMQ_awareness | -0.01 | 0.15 | -0.32 | -0.17 | 0.08 | 0.14 | 0.04 | 0.27 | -0.09 | 0.27 | 0.32 | 0.25 |
| | ΔFFMQ_nonjudging | -0.03 | 0.10 | -0.18 | -0.06 | 0.15 | 0.20 | -0.11 | -0.08 | -0.23 | 0.22 | 0.34 | 0.32 |
| | ΔFFMQ_nonreactivity | 0.04 | 0.16 | -0.20 | 0.05 | 0.18 | 0.28 | 0.07 | 0.06 | -0.24 | 0.06 | 0.25 | 0.28 |
| Breathing | ΔFFMQ_observing | -0.01 | 0.00 | -0.05 | 0.04 | 0.20 | 0.17 | -0.29 | -0.33 | -0.38 | -0.09 | 0.14 | 0.18 |
| | ΔFFMQ_describling | 0.20 | 0.29 | 0.34 | 0.11 | -0.18 | -0.14 | 0.04 | 0.03 | 0.20 | 0.05 | -0.13 | -0.09 |
| | ΔFFMQ_awareness | -0.20 | -0.15 | -0.13 | -0.24 | -0.04 | -0.04 | -0.30 | -0.16 | -0.01 | -0.04 | 0.03 | 0.08 |
| | ΔFFMQ_nonjudging | -0.41 | -0.39 | -0.33 | -0.34 | 0.01 | 0.02 | -0.44 | -0.28 | -0.05 | -0.14 | 0.15 | 0.15 |
| | ΔFFMQ_nonreactivity | -0.36 | -0.36 | -0.46 | -0.42 | -0.06 | 0.04 | -0.42 | -0.38 | -0.24 | -0.26 | 0.01 | 0.13 |
| Body-scan | ΔFFMQ_observing | 0.01 | -0.02 | -0.25 | 0.09 | 0.31 | 0.33 | -0.17 | -0.24 | -0.29 | 0.14 | 0.40 | 0.31 |
| | ΔFFMQ_describling | 0.16 | 0.21 | 0.10 | -0.01 | -0.03 | -0.05 | 0.15 | 0.19 | 0.25 | 0.24 | 0.09 | -0.07 |
| | ΔFFMQ_awareness | -0.32 | -0.26 | -0.29 | -0.39 | -0.01 | 0.17 | 0.04 | 0.08 | 0.08 | 0.16 | 0.32 | 0.19 |
| | ΔFFMQ_nonjudging | -0.37 | -0.36 | -0.43 | -0.47 | -0.04 | 0.23 | -0.26 | -0.20 | -0.19 | -0.09 | 0.24 | 0.25 |
| | ΔFFMQ_nonreactivity | -0.29 | -0.22 | -0.44 | -0.35 | -0.02 | 0.24 | -0.08 | -0.01 | -0.16 | -0.07 | 0.17 | 0.20 |
| Resting 2 | ΔFFMQ_observing | 0.06 | -0.13 | -0.45 | -0.07 | 0.20 | 0.24 | -0.34 | -0.53* | -0.68** | -0.18 | 0.10 | 0.27 |
| | ΔFFMQ_describling | 0.15 | 0.18 | -0.17 | -0.08 | -0.18 | -0.13 | 0.04 | -0.12 | -0.35 | -0.17 | -0.23 | -0.13 |
| | ΔFFMQ_awareness | -0.22 | -0.19 | -0.35 | -0.27 | 0.07 | 0.11 | -0.35 | -0.31 | -0.36 | -0.23 | 0.02 | 0.09 |
| | ΔFFMQ_nonjudging | -0.34 | -0.30 | -0.40 | -0.11 | 0.17 | 0.25 | -0.34 | -0.31 | -0.27 | -0.17 | 0.19 | 0.26 |
| | ΔFFMQ_nonreactivity | -0.20 | -0.18 | -0.51* | -0.31 | 0.04 | 0.19 | -0.37 | -0.44 | -0.54* | -0.41 | -0.06 | 0.12 |

**SUPPLEMENTARY TABLE 3. Correlation coefficients among the power changes of EEG wave bands and DERS subscales in MBSR group.** *$p < .05$, **$p < .01$

| | | Fz | | | | | | Pz | | | | | |
|---|---|---|---|---|---|---|---|---|---|---|---|---|---|
| | | Δdelta | Δtheta | Δalpha | Δlow-beta | Δhigh-beta | Δlow-gamma | Δdelta | Δtheta | Δalpha | Δlow-beta | Δhigh-beta | Δlow-gamma |
| Resting 1 | ΔDERS_nonaccept | 0.16 | 0.09 | 0.24 | 0.12 | -0.15 | -0.37 | 0.06 | 0.05 | -0.06 | -0.33 | -0.45 | -0.43 |
| | ΔDERS_goals | 0.18 | 0.11 | 0.32 | 0.00 | -0.30 | -0.35 | 0.27 | 0.17 | 0.27 | -0.15 | -0.40 | -0.38 |
| | ΔDERS_impulse | 0.16 | 0.02 | 0.29 | 0.18 | -0.05 | -0.16 | 0.10 | 0.00 | 0.12 | -0.21 | -0.30 | -0.27 |
| | ΔDERS_aware | -0.07 | -0.10 | 0.13 | -0.23 | -0.34 | -0.38 | -0.33 | -0.29 | -0.38 | -0.61** | -0.50* | -0.37 |
| | ΔDERS_strategies | -0.03 | -0.12 | -0.01 | -0.02 | -0.22 | -0.29 | -0.22 | -0.17 | -0.19 | -0.48 | -0.46 | -0.39 |
| | ΔDERS_clarity | -0.18 | -0.28 | -0.05 | -0.17 | -0.03 | -0.05 | -0.30 | -0.31 | -0.30 | -0.55** | -0.30 | -0.12 |
| Breathing | ΔDERS_nonaccept | 0.33 | 0.24 | 0.25 | 0.19 | -0.21 | -0.25 | 0.25 | 0.24 | -0.08 | -0.09 | -0.31 | -0.32 |
| | ΔDERS_goals | 0.29 | 0.22 | 0.30 | 0.11 | -0.26 | -0.23 | 0.15 | 0.04 | -0.06 | -0.14 | -0.37 | -0.34 |
| | ΔDERS_impulse | 0.30 | 0.23 | 0.20 | 0.23 | -0.03 | -0.04 | 0.24 | 0.12 | -0.14 | -0.02 | -0.20 | -0.16 |
| | ΔDERS_aware | 0.07 | -0.10 | -0.20 | -0.18 | -0.21 | -0.30 | -0.03 | -0.16 | -0.31 | -0.24 | -0.32 | -0.29 |
| | ΔDERS_strategies | 0.30 | 0.29 | 0.25 | 0.24 | -0.16 | -0.14 | 0.35 | 0.28 | 0.01 | 0.07 | -0.22 | -0.18 |
| | ΔDERS_clarity | -0.12 | -0.18 | -0.23 | -0.22 | -0.11 | 0.02 | -0.07 | -0.13 | -0.33 | -0.31 | -0.16 | -0.03 |
| Body-scan | ΔDERS_nonaccept | 0.21 | 0.19 | 0.21 | 0.23 | -0.22 | -0.40 | 0.11 | 0.15 | 0.11 | -0.05 | -0.34 | -0.35 |
| | ΔDERS_goals | 0.37 | 0.31 | 0.38 | 0.29 | -0.08 | -0.32 | 0.38 | 0.32 | 0.31 | 0.10 | -0.25 | -0.32 |
| | ΔDERS_impulse | 0.34 | 0.30 | 0.31 | 0.44 | 0.07 | -0.18 | 0.16 | 0.13 | 0.09 | 0.07 | -0.21 | -0.21 |
| | ΔDERS_aware | 0.18 | 0.06 | 0.06 | -0.04 | -0.29 | -0.31 | 0.23 | 0.08 | 0.04 | -0.11 | -0.33 | -0.30 |
| | ΔDERS_strategies | 0.14 | 0.12 | 0.14 | 0.11 | -0.27 | -0.36 | -0.05 | -0.04 | -0.08 | -0.22 | -0.44 | -0.32 |
| | ΔDERS_clarity | -0.06 | -0.07 | -0.13 | -0.03 | -0.12 | -0.07 | -0.12 | -0.03 | -0.14 | -0.27 | -0.25 | -0.07 |
| Resting 2 | ΔDERS_nonaccept | 0.24 | 0.15 | 0.15 | -0.04 | -0.37 | -0.40 | 0.22 | 0.13 | 0.11 | -0.14 | -0.32 | -0.34 |
| | ΔDERS_goals | 0.51* | 0.37 | 0.27 | -0.08 | -0.32 | -0.33 | 0.36 | 0.20 | 0.14 | -0.05 | -0.32 | -0.37 |
| | ΔDERS_impulse | 0.38 | 0.19 | 0.15 | 0.04 | -0.18 | -0.21 | 0.33 | 0.14 | 0.10 | 0.02 | -0.21 | -0.22 |
| | ΔDERS_aware | 0.27 | 0.07 | 0.01 | -0.22 | -0.33 | -0.31 | 0.20 | 0.13 | 0.19 | -0.13 | -0.23 | -0.32 |
| | ΔDERS_strategies | 0.27 | 0.21 | 0.24 | 0.09 | -0.24 | -0.26 | 0.23 | 0.16 | 0.15 | 0.02 | -0.29 | -0.25 |
| | ΔDERS_clarity | 0.14 | -0.03 | -0.05 | -0.25 | -0.17 | -0.06 | -0.03 | -0.13 | -0.08 | -0.30 | -0.20 | -0.07 |

## 3 Reference

Isbel, B., Lagopoulos, J., Hermens, D. F., & Summers, M. J. (2019). Mindfulness induces changes in anterior alpha asymmetry in healthy older adults. *Mindfulness*, *10*(7), 1381-1394. https://doi.org/10.1007/s12671-019-01106-w
